# Nucleic Acid Therapeutics for “Undruggable” Cancer Targets: Mechanisms, Challenges, and Prospects

**DOI:** 10.1002/advs.75837

**Published:** 2026-05-28

**Authors:** Feng Xu, Ke Wang, Kaizhong Lu, Tianlun Hou, Yuan Chen, Xian Wang, Hongchuan Jin

**Affiliations:** ^1^ Department of Medical Oncology Sir Run Run Shaw Hospital School of Medicine Zhejiang University Hangzhou Zhejiang P. R. China; ^2^ Department of Oncology The First Affiliated Hospital with Nanjing Medical University Nanjing Jiangsu P. R. China

**Keywords:** Nucleic acid therapeutics, undruggable targets, MYC, p53, Ras

## Abstract

Despite significant advances in precision oncology, key oncoproteins such as Ras, MYC, and p53 remain historically difficult to drug. Conventional pharmacologic strategies are fundamentally constrained by their dependency on direct interactions with well‐defined structural domains, which these targets lack. Nucleic acid therapeutics offer a transformative paradigm to overcome this central limitation by redirecting pharmacological intervention to the mRNA and genomic levels, thereby operating independently of complex protein structures. In this review, we systematically examine the mechanisms of action and translational progress of diverse nucleic acid modalities, including ASOs, siRNAs, miRNAs, aptamers, and mRNA vaccines against these intractable targets. We comprehensively discuss their mechanisms, such as transcript degradation, translational inhibition, and upstream regulatory interference. Furthermore, we critically analyze the primary translational bottlenecks, specifically focusing on delivery efficiency, safety profiling, and scalable manufacturing, while highlighting recent advances in nanocarrier platforms. Finally, we explore future directions enabled by emerging technologies and computational design. Ultimately, this review highlights how nucleic acid therapeutics represent a paradigm shift, enabling precise regulation of “undruggable” cancer targets.

## Graphical abstract

Nucleic acid therapeutics bypass the structural limitations of conventional drugs by targeting mRNA rather than proteins. This review examines how antisense oligonucleotides, siRNAs, miRNAs, aptamers, and mRNA vaccines intervene against historically undruggable oncoproteins including Ras, MYC, and p53, highlighting mechanistic advances, delivery innovations, and translational challenges that are reshaping the frontiers of precision oncology.

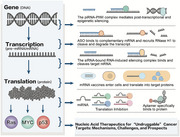

**Abbreviations**
4E‐BP1: eIF4E‐binding protein 1
α‐L‐LNA: alpha‐L‐Locked Nucleic Acid
AI: Artificial Intelligence
Ago2: Argonaute‐2
ASO: Antisense Oligonucleotide
ATM: Ataxia Telangiectasia Mutated
cGAMP: Cyclic GMP‐AMP
circRNA: Circular RNA
CpG: Cytosine‐phosphate‐Guanine
CRISPR: Clustered Regularly Interspaced Short Palindromic Repeats
EGFR: Epidermal Growth Factor Receptor
ERK: Extracellular Signal‐Regulated Kinase
GalNAc: N‐Acetylgalactosamine
GAP: GTPase‐Activating Protein
GEF: Guanine Nucleotide Exchange Factor
GOF: Gain‐of‐Function
GRP78: Glucose‐Regulated Protein 78
HDAC1: Histone Deacetylase 1
HIF1A‐As2: HIF1A Antisense RNA 2
hnRNP H/F: heterogeneous nuclear ribonucleoprotein H and F
HPV16 E6: Human Papillomavirus Type 16 Early Protein 6
HRAS: Harvey Rat Sarcoma virus oncogene homolog
KRAS: Kirsten Rat Sarcoma virus oncogene homolog
LNP: Lipid Nanoparticle
MAPK: Mitogen‐Activated Protein Kinase
MAX: MYC‐Associated Factor X
MDR1: Multidrug Resistance Protein 1
MDM2/4: Mouse Double Minute 2/4
MEK: MAPK/ERK Kinase
miRNA: MicroRNA
mTOR: Mammalian Target of Rapamycin
MYC: Myelocytomatosis oncogene
NRAS: Neuroblastoma Rat Sarcoma virus oncogene homolog
PI3K: Phosphoinositide 3‐Kinase
piRNA: Piwi‐interacting RNA
PIWI: P‐element Induced Wimpy testis
PROTAC: Proteolysis‐Targeting Chimera
PTGS: Post‐Transcriptional Gene Silencing
Ras: Rat Sarcoma virus oncogene homolog
RISC: RNA‐Induced Silencing Complex
RNase H1: Ribonuclease H1
SELEX: Systematic Evolution of Ligands by Exponential Enrichment
siRNA: Small Interfering RNA
SLC7A11: Solute Carrier Family 7 Member 11
STING: Stimulator of Interferon Genes
TCTP: Translationally Controlled Tumor Protein
TNA: Threose Nucleic Acid
TP53: Tumor Protein 53
UTR: Untranslated Region
VEGF: Vascular Endothelial Growth Factor

## 1 Introduction

Throughout the history of drug discovery, a persistent challenge has been the existence of disease driving‐proteins that lack conventional binding sites for small molecules or antibodies [1]. These so‐called undruggable targets often feature smooth surfaces devoid of hydrophobic pockets or rely on extensive protein‐protein interaction interfaces, rendering traditional therapeutic approaches ineffective [2]. Among these, canonical oncoproteins, including the GTPase rat sarcoma virus oncogene homolog (Ras), the transcription factor myelocytomatosis oncogene (MYC), and the tumor suppressor tumor protein 53 (p53) are prime therapeutic targets [3, 4, 5]. These proteins are frequently mutated or functionally dysregulated in human cancers, driving tumor initiation, progression, and therapeutic resistance. However, despite decades of intensive effort, direct targeting therapies against them have made limited clinical progress, representing a major bottleneck in cancer treatment that demands innovative solutions [6].

The fundamental limitation of conventional drug paradigms lies in their reliance on lock and key binding to 3D protein structures [7]. Small molecule inhibitors and monoclonal antibodies, despite their clinical success against many targets, are inherently ill suited for proteins lacking well‐defined binding pockets or those functioning through dynamic protein–protein interactions. This dilemma has created an urgent need for therapeutic strategies that can circumvent the requirement for direct protein binding [8]. Nucleic acid therapeutics offer precisely such a transformative solution [9]. By operating at the mRNA level through Watson‐Crick base pairing rules, modalities including antisense oligonucleotides (ASOs), small interfering RNA (siRNA), and mRNA‐based drugs enable sequence‐specific regulation of gene expression before the target protein is even synthesized. This fundamental shift in intervention level transforms the challenge from targeting an undruggable protein to addressing a targetable genetic sequence, thereby opening new avenues for precision oncology [10].

**FIGURE 1:**
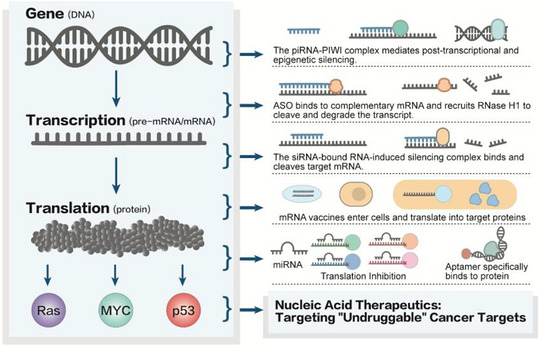
Central Dogma‐Based Nucleic Acid Approaches to “Undruggable” Oncoproteins. The schematic illustrates central dogma‐based targeting of classic undruggable oncoproteins (Ras, MYC, p53) by nucleic acid therapeutics.

Building on this rationale, this review provides a systematic overview of nucleic acid therapeutics targeting undruggable oncoproteins, as illustrated in Figure 1. We first describe the major classes of nucleic acid therapeutics, including ASOs, siRNA, miRNA, aptamers, piRNA, and mRNA, along with their respective mechanisms of action, such as transcript degradation, translational suppression, splicing modulation, and upstream regulatory interference. Using Ras, MYC, and p53 as representative paradigms, we then summarize preclinical and clinical progress for each target across different nucleic acid modalities. We critically analyze the core challenges impeding clinical translation, with an emphasis on delivery efficiency, safety concerns, and manufacturing scalability, while discussing recent advances in nanocarrier platforms. Finally, we highlight emerging technologies, including circular RNA (circRNA), mRNA protein replacement, CRISPR‐based gene editing, and artificial intelligence (AI)‐driven design, and we offer our perspective on how these innovations may further redefine druggability in oncology.

## 2 Types and Mechanisms of Action of Nucleic Acid Therapeutics

### 2.1 ASOs

#### 2.1.1 Mechanisms of Action

ASOs are short, single‐stranded oligonucleotides, typically 13–15 nucleotides in length, that bind specifically to target RNA via Watson‐Crick base‐pairing rules [11]. ASOs can modulate gene expression through RNase H1‐dependent mRNA degradation, translation blockade, and pre‐mRNA splicing regulation. The RNase H1‐dependent mechanism is the most classical mode of action. Upon forming a DNA‐RNA heteroduplex with the target mRNA, ASOs recruit the endogenous RNase H1 enzyme, leading to specific cleavage and degradation of the target mRNA [12, 13, 14]. Additionally, they can inhibit ribosomal binding through a steric hindrance effect [15]. Furthermore, ASOs can be designed to bind specific splice sites or regulatory elements in pre‐mRNA, redirecting splicing to alter exon inclusion patterns, thereby modulating the expression of specific protein isoforms [16]. These mechanisms collectively enable ASOs to precisely regulate gene expression at the post‐transcriptional level, offering a novel intervention strategy for cancer therapy [17, 18, 19].

By operating at the RNA level, ASOs provide a fundamentally different approach to targeting traditionally “undruggable” oncoproteins. Many oncogenic products are difficult to target with small molecules or antibodies due to their lack of typical druggable binding pockets. ASOs overcome this limitation by targeting the RNA transcripts encoding these proteins. They achieve functional silencing or correction by degrading the target mRNA or altering its splicing to produce loss‐of‐function isoforms. Furthermore, computationally designed ASOs enable the rational targeting of previously non‐intervenable oncogenic RNAs and can correct the abnormal expression of disease‐causing genes through splice‐switching technology. This opens new avenues in cancer therapy for targeting “undruggable” oncoproteins [20]. Currently, ASOs have demonstrated potential in areas such as targeted cancer therapy and modulation of the tumor immune microenvironment, yet their clinical translation remains constrained by challenges related to delivery efficiency, safety, and immunogenicity [21, 22].

#### 2.1.2 Case Study

The clinical feasibility of ASO‐based targeting of undruggable oncoproteins was first validated by Cunningham and colleagues in a Phase I clinical trial of ISIS 2503, an ASO targeting HRAS, which demonstrated favorable safety profiles and disease stabilization in patients with advanced solid tumors [23]. More recently, Ross and colleagues developed AZD4785, a high‐affinity ASO targeting KRAS, which reduced KRAS expression, suppressed MAPK signaling, and induced tumor regression in non‐small cell lung cancer xenograft models [24].

### 2.2 SiRNA

#### 2.2.1 Mechanisms of Action

siRNA is a short double‐stranded RNA molecule that mediates sequence‐specific degradation of target mRNA through the RNA interference pathway, thereby silencing the expression of oncogenes or tumor‐associated genes [25]. The core mechanism involves loading of the siRNA duplex into the RNA‐induced silencing complex (RISC). Within RISC, the passenger strand is removed, and the guide strand directs sequence‐specific recognition of complementary mRNA. The endonuclease activity of Argonaute‐2 (Ago2) then cleaves the target mRNA, leading to its degradation and sustained suppression of gene expression [26, 27, 28]. Owing to its high sequence specificity, siRNA has been widely explored in cancer therapy to suppress oncogenic signaling pathways or enhance antitumor responses.

By acting at the mRNA level, siRNA provides a powerful alternative for targeting traditionally “undruggable” proteins. Unlike conventional drugs that require binding to specific structural domains of proteins, siRNA operates at the mRNA level through Watson‐Crick base‐pairing rules, enabling it to theoretically target any gene transcript with a known sequence. This capability allows siRNA to effectively silence proteins traditionally deemed “undruggable” due to the absence of druggable pockets, such as transcription factors, refractory oncoproteins, and non‐enzymatic scaffolding proteins in signaling pathways [29, 30, 31, 32, 33]. Moreover, siRNA can be employed to overcome tumor drug resistance. For instance, silencing multidrug resistance protein 1 (MDR1) or anti‐apoptotic genes can restore tumor cell sensitivity to chemotherapy or targeted therapies [34, 35, 36, 37]. In contrast to small molecules, siRNA avoids the need for protein binding sites by targeting mRNA, thereby halting pathogenic protein synthesis at its source.

In cancer therapy, siRNA has been used to target key oncogenes, reverse drug resistance, and remodel the tumor immune microenvironment [38, 39, 40, 41, 42]. However, its clinical translation faces challenges such as inefficient delivery, off‐target effects, and poor in vivo stability. Currently, ongoing efforts are focused on addressing these bottlenecks through advanced delivery systems such as lipid nanoparticles (LNPs) and chemical modification strategies, aiming to fully realize its potential in targeting “undruggable” therapeutic targets [43].

#### 2.2.2 Case Study

Yuan and colleagues developed a combinatorial siRNA library targeting multiple nodes of the Ras‐MAPK signaling pathway and demonstrated effective tumor growth suppression in colorectal cancer models [31]. In pancreatic cancer, Anthiya and colleagues employed tLyp‐1 peptide modified lipid nanoparticles for the targeted delivery of anti‐KRAS siRNA, which significantly enhanced the efficacy of gemcitabine [32].

### 2.3 MiRNAs

#### 2.3.1 Mechanisms of Action

MicroRNAs (miRNAs) are a class of endogenous non‐coding RNAs, approximately 22 nucleotides in length, which finely regulate gene expression networks at the post‐transcriptional level by incompletely base‐pairing with the 3′‐untranslated regions (3′‐UTRs) of target mRNAs [44, 45]. Unlike the high specificity of siRNA, a single miRNA can simultaneously regulate dozens to hundreds of downstream genes, forming complex regulatory networks [46].

From the perspective of “undruggable” targets, miRNA therapeutics offer a unique network‐based intervention strategy. The initiation and progression of tumors are typically not driven by a single gene but involve the dysregulation of complex, multi‐pathway networks. This complexity often limits the efficacy of conventional drugs that target individual proteins. miRNA therapy addresses this challenge by restoring key tumor‐suppressive miRNAs or inhibiting oncogenic miRNAs, enabling coordinated modulation of entire disease‐driving signaling networks and thereby overcoming the limitations of single‐target approaches [47, 48]. This ability to simultaneously regulate multiple genes and pathways makes miRNA a powerful tool for intervening in complex disease phenotypes and signaling networks [49, 50, 51, 52, 53]. Despite persistent challenges in delivery and safety, these agents have emerged as a key research direction in cancer treatment owing to their capacity to simultaneously modulate multiple tumor‐related pathways.

#### 2.3.2 Case Study

Pramanik and colleagues demonstrated that systemic delivery of tumor suppressor miRNAs miR‐143, miR‐145, and let‐7 using lipid nanoparticles effectively inhibited pancreatic cancer growth in mouse models [54]. Ibrahim and colleagues showed that polyethylenimine‐mediated delivery of unmodified miR‐145 suppressed tumor growth in a colon cancer mouse model accompanied by c‐Myc downregulation and enhanced apoptosis [55].

### 2.4 Aptamers

#### 2.4.1 Mechanisms of Action

Aptamers are single‐stranded nucleic acid molecules obtained through in vitro selection techniques, which fold into unique 3D structures capable of binding to their targets with high affinity and specificity [56, 57]. Its mechanism involves directly blocking the function of target proteins, modulating their activity, and serving as “smart carriers” to guide therapeutic agents for targeted delivery to disease sites [56, 58].

Aptamer technology offers a novel paradigm for targeting and regulating “undruggable” targets [59, 60]. Conventional drug development is constrained by the requirement for target proteins to possess well‐defined active pockets or binding interfaces. In contrast, aptamers use their structural plasticity to recognize and tightly bind to traditionally “undruggable” protein surfaces, offering a strategy for functional inhibition or allosteric modulation [61]. This characteristic enables aptamers to intervene in proteins lacking enzymatic activity or canonical binding domains, such as transcription factors and scaffolding proteins, thereby broadening the spectrum of targetable therapeutic interfaces.

In cancer therapy, aptamers have been widely applied in constructing targeted delivery systems and regulating the tumor immune microenvironment [62, 63, 64, 65, 66, 67]. Despite challenges in vivo stability, manufacturing, and clinical translation, aptamers have emerged as a critical bridge to “undruggable” targets, offering programmable design, low immunogenicity, and unique capacity to bind “undruggable” protein surfaces [68, 69, 70, 71].

#### 2.4.2 Case Study

Wang and colleagues engineered a PROTAC molecule named ProMyc based on the c‐Myc binding aptamer MA9C1, which recruits E3 ubiquitin ligases to ubiquitinate and degrade c‐Myc, leading to significant tumor regression in animal models [72]. For mutant p53, Kong and colleagues rationally engineered a DNA aptamer that directly binds the p53‐R175H hotspot mutant and developed a PROTAC‐based degrader capable of specifically inducing degradation of the oncogenic mutant protein [73].

### 2.5 PiRNA

#### 2.5.1 Mechanisms of Action

Piwi‐interacting RNA (piRNAs) are a class of predominantly P‐element Induced WImpy testis (PIWI)‐associated non‐coding single‐stranded RNAs. Their maturation process is independent of Dicer and is primarily completed through processing by the PIWI protein complex [74, 75, 76]. The mature piRNA, in complex with PIWI proteins (forming the piRISC), not only recognizes and cleaves complementary transposon RNAs to maintain genomic stability but can also recruit epigenetic modifiers or regulate specific mRNAs at the post‐transcriptional level, thereby executing multi‐layered gene silencing functions [77, 78, 79].

From the perspective of targeted intervention, piRNA and its complexes offer a new dimension for targeting “undruggable” regulatory networks [80]. The occurrence of many tumors is associated with global and mechanistically complex pathological processes, such as epigenetic dysregulation and aberrant transposon activation. These processes often lack traditional single‐protein targets. The piRNA system, an endogenous epigenetic and post‐transcriptional hub, along with its key functional nodes, thus represents a network of novel targets [81, 82, 83]. The use of ASOs or piRNA mimics to modulate oncogenic pathways offers a route to target dysregulated networks in chromatin and genome stability, bypassing the limitations of small‐molecule drugs [84, 85, 86, 87, 88].

Despite their aberrant expression and therapeutic potential in cancer, the clinical translation of piRNAs remains nascent, as it faces challenges such as complex mechanisms, inefficient delivery, and tissue specificity [83, 89]. Nevertheless, the unique functions of piRNA and its critical role in tumorigenesis are driving its translation from basic research to clinical application, promising novel precision cancer therapies.

#### 2.5.2 Case Study

Wang and colleagues discovered that in lung squamous cell carcinoma, piR‐L‐138 directly binds to and inhibits p60‐MDM2, a key negative regulator of p53, thereby impairing p53‐mediated apoptosis and inducing resistance to cisplatin chemotherapy [90]. This finding provides a novel intervention target for overcoming drug resistance.

### 2.6 MRNA

#### 2.6.1 Mechanisms of Action

mRNA therapeutics utilize chemically modified and optimized in vitro transcribed mRNA, which is delivered into host cells via systems such as LNPs. Upon cellular entry, it directly guides the production of functional proteins, thereby facilitating the compensation of deficient proteins or the expression of therapeutic antigens [91, 92].

Targeting “undruggable” oncoproteins, mRNA‐based therapy provides a replacement strategy [93]. mRNA delivery circumvents the challenge of direct protein targeting, providing a viable approach for loss‐of‐function disorders and targets constrained by structural or functional limitations [94, 95]. mRNA therapy overcomes target undruggability by restoring functional protein expression intracellularly, thereby addressing the core pathological deficiency [96, 97, 98].

In cancer therapy, research on mRNA vaccines primarily focuses on using mRNA sequences that encode tumor‐specific antigens or modulate the anti‐tumor capacity of immune cells to elicit a personalized anti‐tumor immune response [99]. Currently, this field is focused on enhancing efficacy through optimizing neoantigen prediction algorithms, improving LNP delivery systems, and exploring combination therapies with immune checkpoint inhibitors [100, 101, 102, 103, 104, 105, 106]. mRNA technology faces multiple challenges, including the accuracy of neoantigen screening, the immunosuppressive tumor microenvironment, and the complexities of personalized manufacturing. Nevertheless, with its flexible design, rapid development, and capacity for intracellular protein synthesis, it offers a promising strategy to address “undruggable” targets and tumor heterogeneity [107, 108, 109].

#### 2.6.2 Case Study

Cafri and colleagues demonstrated that personalized mRNA vaccines could induce KRAS G12D‐specific T cell responses in patients with gastrointestinal cancer, providing critical evidence for combination immunotherapy [110]. Wang and colleagues further confirmed that an mRNA vaccine targeting the KRAS G12V mutation, when combined with the PD‐1 inhibitor pembrolizumab, produced clinical benefit in patients with advanced solid tumors [111].

Table 1 provides a systematic comparison of the six nucleic acid modalities discussed above, summarizing their mechanisms, target suitability, delivery requirements, duration of action, clinical stage, and key limitations.

**TABLE 1:** Comparison of nucleic acid therapeutic modalities for undruggable targets.

MODALITY	MECHANISM	TARGET TYPE SUITABILITY	DELIVERY REQUIREMENT	DURATION	CLINICAL STAGE FOR Ras/MYC/p53	Key Limitation
ASO	mRNA degradation, splicing modulation	Any transcript	Chemical modification, LNP/GalNAc	Weeks	Phase I/II (ISIS 2503, AZD4785)	RNase H1 dependent
siRNA	RISC mediated mRNA cleavage	Any transcript	LNP, GalNAc, nanoparticle	Weeks	Preclinical to Phase I	Endosomal escape
miRNA	Network regulation (multiple targets)	Multi‐pathway networks	LNP	Weeks	Preclinical	Off‐target complexity
Aptamer	Protein binding, PROTAC	Proteins lacking pockets	No special carrier	Days to weeks	Preclinical (ProMyc, dp53m)	In vivo stability
piRNA	Epigenetic, post‐transcriptional	Regulatory networks	LNP	Unknown	Early preclinical	Mechanism unclear
mRNA	Protein replacement, vaccination	Loss‐of‐function, antigens	LNP	Days	Phase I (KRAS vaccines)	Immunogenicity

## 3 Typical “Undruggable” Targets: Ras, MYC, and p53

### 3.1 Ras

The Ras family is one of the most frequently activated proto‐oncogene families in human cancers, primarily including Harvey Rat Sarcoma virus oncogene homolog (HRAS), Neuroblastoma Rat Sarcoma virus oncogene homolog (NRAS), and Kirsten Rat Sarcoma virus oncogene homolog (KRAS) [112]. Ras proteins are small GTPases that operate on the inner surface of the plasma membrane, where they function as crucial molecular switches [113]. It cycles between an active state (GTP‐bound) and an inactive state (GDP‐bound), precisely regulating several vital intracellular signaling pathways, such as the RAF‐MEK‐ERK (MAPK) pathway and the PI3K‐AKT‐mTOR pathway [114]. These pathways directly influence critical cellular processes, including proliferation, differentiation, survival, and metabolism [115]. Mutations in the Ras gene result in its protein being permanently locked in the constitutively activated state, leading to the continuous transmission of pro‐proliferative and anti‐apoptotic signals downstream, thereby driving tumor initiation and progression. Statistics indicate that Ras mutations are present in approximately 20% of all human malignancies, with extremely high mutation rates particularly in pancreatic, colorectal, and lung cancers, making it one of the most critical targets in oncology research [116].

#### 3.1.1 Mechanisms of “Undruggable”

**FIGURE 2:**
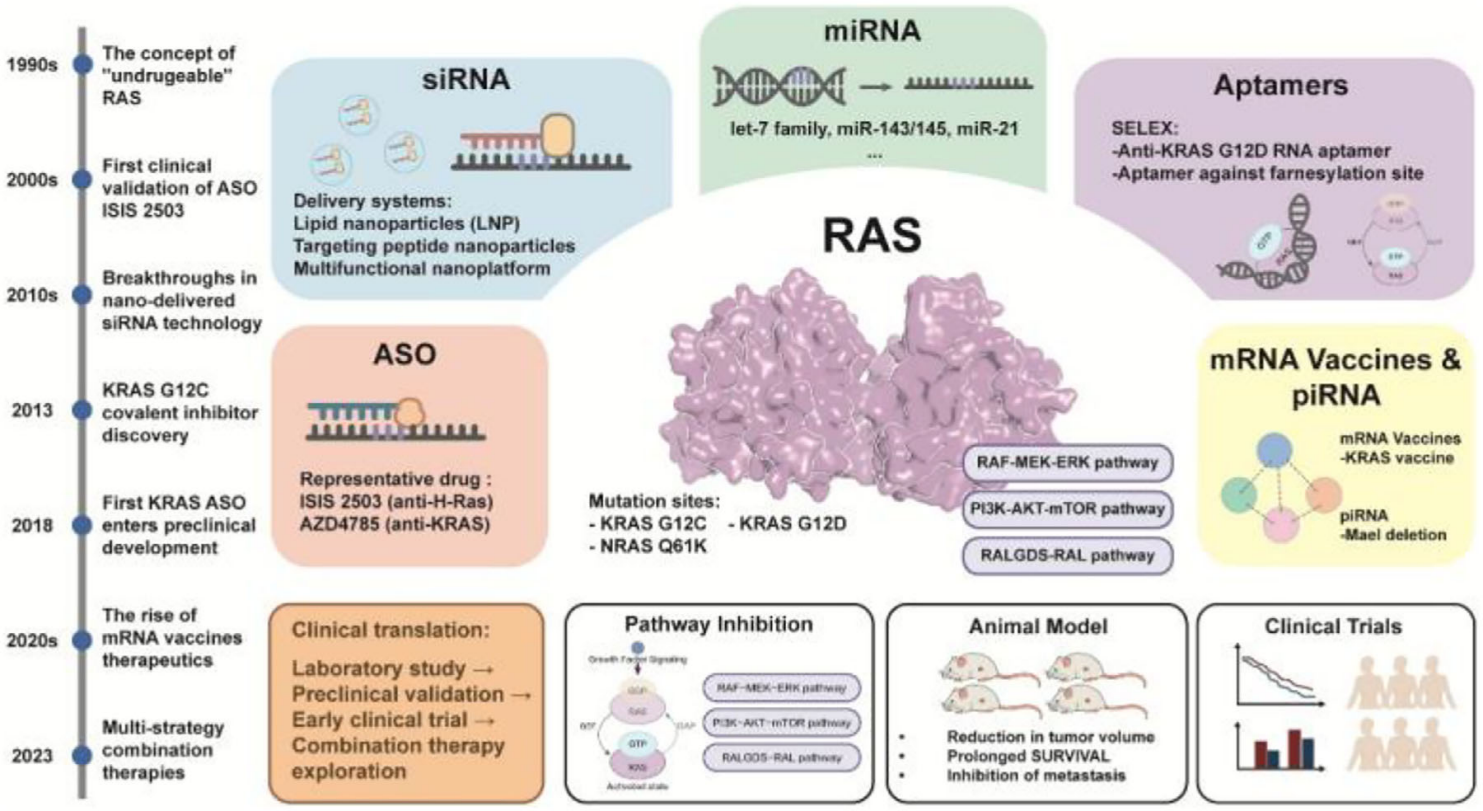
Ras Oncoprotein: The Evolution of Nucleic Acid Therapeutics. This schematic chronologically illustrates the development of diverse nucleic acid strategies against the Ras target, covering ASOs, siRNAs, miRNAs, aptamers, mRNA vaccines, and piRNAs. The Ras protein structure image was generated with MolViz (https://molviz.com).

For decades, Ras has been considered an “undruggable” target, primarily due to two core mechanisms. First, it exhibits an extremely high binding affinity for GTP/GDP at the picomolar level, making it nearly impossible for endogenous small molecules to competitively displace them. Second, its protein surface is smooth and lacks well‐defined pockets suitable for high‐affinity drug binding [2]. Moreover, the active surface of Ras proteins is relatively smooth and lacks well‐defined binding sites that would allow traditional small‐molecule inhibitors to engage with high affinity [117]. This renders the rational design of competitive inhibitors capable of effectively abrogating the interaction between Ras‐GTP and its downstream effector proteins extremely challenging. Another major hurdle lies in the absence of well‐defined allosteric regulatory sites. Conventional drug design strategies typically capitalize on protein allosteric sites, which are regions distinct from the active site yet capable of modulating protein functions [118]. Nevertheless, in early studies, Ras proteins were also regarded as lacking suitable allosteric pockets amenable to drug development. Their conformational changes are primarily governed by large macromolecules such as guanine nucleotide exchange factors (GEFs) and GTPase‐activating proteins (GAPs), which are thus difficult to mimic or perturb using small molecules [119]. Despite these formidable challenges, persistent efforts by researchers in recent years have yielded revolutionary breakthroughs in the field of Ras‐targeted therapy, including the direct targeting of KRAS G12C mutants and the indirect targeting of downstream signaling pathways [120, 121]. Among these advances, nucleic acid‐based therapeutics have achieved considerable progress (Figure 2).

#### 3.1.2 ASOs

In ASO‐based therapeutic strategies targeting Ras, multiple preclinical and clinical studies have corroborated the efficacy and application potential of such regimens for Ras targeting. In early research, a phase I clinical trial conducted by Cunningham et al. demonstrated that ISIS 2503, an ASO against HRAS, exhibited favorable safety profiles in patients with advanced solid tumors and could induce disease stabilization, thus providing the first clinical validation of the feasibility of Ras inhibition strategies [23]. Morse's technical evaluation further indicated that this agent has become the first Ras‐targeting oligonucleotide to enter systematic clinical validation and has been advanced to phase II clinical trials [122]. Mechanistically, Ross et al. identified an ASO capable of selectively silencing mutant KRAS via rational screening. This ASO not only abrogated the ERK signaling pathway but also suppressed the secretion of vascular endothelial growth factor‐A (VEGF‐A), thereby suggesting that Ras inhibition may confer an anti‐angiogenic effect [123]. In various animal models, ASOs have also exhibited prominent antitumor efficacy. Wickstrom et al. demonstrated that ASOs targeting HRAS and KRAS led to an 80% and 50% reduction in the volume of xenografted bladder cancer and pancreatic cancer tumors, respectively, with the therapeutic effect being durable [124]. Morioka et al. further validated in a hamster model of pancreatic cancer that KRAS‐targeting ASOs exerted consistent and potent inhibitory effects on primary tumors, metastatic lesions, and even recurrent metastatic foci, with efficacy superior to that of conventional chemotherapeutic agents [125]. In terms of technological advancements, Fluiter et al. developed a gapmer‐type ASO containing α‐L‐locked nucleic acid (α‐L‐LNA), which could potently knock down HRAS mRNA and suppress tumor growth at an ultralow dose, with no obvious toxicity observed [126]. In recent years, antisense‐based therapeutic strategies have continued to advance. For instance, Ross et al. reported the high‐affinity ASO AZD4785, which, in non‐small cell lung cancer xenograft models, reduced KRAS expression, suppressed MAPK signaling, and induced tumor regression [24]. This agent is under preclinical development. In a broader context, Saxena et al. reviewed that parallel advances are being made with ASO strategies targeting Ras membrane localization, downstream signaling, or expression itself, outlining key therapeutic directions [127].

#### 3.1.3 SiRNA

In siRNA‐based therapeutic strategies targeting KRAS, nanoparticle‐mediated siRNA delivery has exhibited remarkable therapeutic potential and application versatility. Multiple studies have established functionalized nanosystems to achieve efficient silencing of KRAS and elicit synergistic antitumor effects. Specifically, Lin et al. and Zhang et al. encapsulated KRAS siRNA using biodegradable polyester nanoparticles and carbon dioxide‐derived cationic polycarbonate nanoparticles, respectively. These nanocomplexes effectively downregulated target gene expression and suppressed tumor proliferation and invasion in pancreatic cancer models [128, 129]. Yang et al. developed BCPV nanoparticles, while another research team led by Yang proposed the conjugated oligolectrolyte nanocomplex COE‐S6/siRNA. Both nanosystems exhibited high transfection efficiency, favorable safety profiles, and potent inhibition of the KRAS signaling pathway in both in vitro and in vivo models [130, 131]. To improve targeting specificity and therapeutic efficacy, a variety of active targeting and combination strategies have been widely adopted. Anthiya et al. employed tLyp‐1 peptide‐modified LNPs for the targeted delivery of anti‐KRAS siRNA, which significantly enhanced the efficacy of gemcitabine in pancreatic cancer models [32]. Peptide‐based nanoparticles developed by Strand et al. can precisely target KRAS‐mutant tumors, achieving specific gene silencing and halting tumor progression [132]. In terms of combination therapy, Yin et al. leveraged functionalized graphene oxide for the co‐delivery of HDAC1 and KRAS G12C siRNAs, and the combined treatment with photothermal therapy resulted in over 80% tumor regression [133]. Kim et al. employed tGC nanoparticles to co‐deliver siKRAS and PI3K inhibitors, which elicited a synergistic antitumor effect in ovarian cancer harboring KRAS/PTEN mutations [134]. Furthermore, Yuan et al. constructed a combinatorial siRNA library targeting multiple nodes of the Ras‐MAPK signaling pathway, which effectively suppressed tumor growth in colorectal cancer models [31], and a subsequent Phase II trial of a KRAS G12D/V‐targeting siRNA implant (siG12D‐LODER) with chemotherapy has provided clinical proof‐of‐concept in locally advanced pancreatic cancer [135]. In terms of delivery technologies, besides nanomaterials, physical methods such as electroporation have also been successfully applied. Réjiba et al. delivered KRAS siRNA in combination with gemcitabine via electroporation, which significantly prolonged the survival time of orthotopic pancreatic cancer models [136]. On the other hand, viral vectors have also demonstrated great potential. Zhang et al. utilized a recombinant oncolytic adenovirus carrying KRAS siRNA, which achieved potent antitumor effects through the dual mechanisms of viral oncolysis and gene silencing [137]. Collectively, these advances demonstrate that siRNA therapies based on nanocarriers, and diverse delivery systems have emerged as a pivotal strategy for inhibiting oncogenic Ras, and they exhibit broad prospects in combination therapy, precision intervention, and indication expansion.

#### 3.1.4 miRNA

In addition to strategies based on siRNA and ASO, miRNAs have garnered widespread attention in targeting the Ras pathway for cancer therapy due to their ability to simultaneously regulate multiple oncogenic signaling nodes. Numerous studies have shown that various miRNAs play important tumor‐suppressive roles by directly or indirectly targeting Ras and its upstream or downstream molecules. The let‐7 family and miR‐143/145 are widely recognized as direct negative regulators of KRAS. The let 7 can inhibit the expression of KRAS by binding to the 3’UTR of its mRNA, thereby suppressing proliferation and enhancing therapeutic sensitivity in various cancer models. In colorectal cancer, let‐7a enhances sensitivity to chemotherapy and radiotherapy, and this effect is modulated by TP53 status [138]. Furthermore, let‐7 can also inhibit the proliferation and radio resistance of glioma [139]. Similarly, miR‐143 significantly inhibits cell growth and invasion by directly suppressing KRAS expression in colorectal cancer [140] and gastric cancer [141]. miR‐145 also exerts tumor‐suppressive effects by downregulating Ras in gastric cancer [142] and in prostate cancer bone metastasis models [143]. Furthermore, other miRNAs such as miR‐18a [144], miR‐30a [145], miR‐27a‐5p [146], and miR‐708 [147] have also been demonstrated to effectively inhibit the Ras signaling pathway. miR‐377 promotes gastric cancer invasion by inhibiting RASSF8, a negative regulator of Ras, thereby relieving its suppression on Ras [148]. Conversely, miR‐182 promotes oral cancer progression by inhibiting RASA1 and SPRED1 (Ras GAPs and pathway suppressors), thereby activating the MEK‐ERK pathway [149]. Studies have also found that miRNAs can synergistically regulate the Ras network. For example, miR‐21 and let‐7 co‐regulate the KRAS pathway in lung cancer, where combined inhibition yields more significant effects than individual interventions [150]. Notably, some miRNAs may enhance the activity of the Ras signaling pathway. For example, miR‐425‐5p is highly expressed in KRAS‐mutant colorectal cancer and can amplify the Ras/MAPK pathway, which may be associated with resistance to EGFR‐targeted therapies [151]. Moreover, clinical studies have revealed that high expression of miR‐143 is associated with lymph node metastasis and poor prognosis in pancreatic cancer, suggesting its function may be complex and context‐dependent [152]. Multiple studies are dedicated to developing miRNA‐based replacement therapies. Karmakar et al. proposed replacement therapy using miR‐143/145 and let‐7 to inhibit the progression of Ras‐driven tumors [153]. Corresponding delivery strategies are also under development, such as using electroporation to deliver miRNA expression plasmids [154], LNPs to restore miR‐143/145 or miR‐34a expression [54], and constructing single‐molecule dual‐miRNA (let‐7c/miR‐124) nanocarriers to simultaneously inhibit multiple targets [155]. These strategies have demonstrated significant tumor‐suppressive effects in preclinical models. Furthermore, natural compounds such as Oleacein, an olive oil polyphenol, can indirectly suppress oncogenic proteins like KRAS by upregulating tumor‐suppressive miRNAs, such as miR‐193a‐3p and miR‐16 [156]. In summary, modulating the Ras signaling network by restoring or suppressing the function of specific miRNAs has emerged as a promising therapeutic strategy. Their multi‐target properties help overcome tumor compensatory mechanisms and drug resistance, offering new directions for future combination therapies.

#### 3.1.5 Aptamers

In recent years, aptamers have demonstrated significant potential in targeting Ras proteins and modulating their downstream signaling pathways, offering a unique mechanism of action in cancer management. Unlike traditional small molecule inhibitors that require well defined binding pockets, aptamers bind to Ras through structural plasticity, enabling functional inhibition or allosteric modulation. In the context of cancer management, aptamers serve three primary roles. First, they act as direct inhibitors by binding to Ras or its effector binding domains, blocking downstream signal transduction. Second, they function as targeting ligands for precision delivery of chemotherapeutic agents or nanoparticles to Ras driven tumors. Third, aptamer‐based PROTACs enable targeted degradation of Ras proteins, representing a novel modality for eliminating otherwise undruggable oncoproteins.

Jeong et al. utilized systematic evolution of ligands by exponential enrichment (SELEX) technology to select RNA aptamers that specifically recognize the KRAS G12D mutant, providing a highly selective diagnostic and therapeutic tool for tumors driven by mutant KRAS [157]. In contrast, Tanaka et al. employed an improved SELEX method to obtain RNA aptamers targeting the KRAS farnesylation site, thereby blocking its membrane localization and subsequent signal transduction [158]. In the direct inhibition of Ras effector pathways, the peptide aptamer developed by Xu and Luo can distinguish HRAS mutants and effectively block the Ras‐Raf interaction, demonstrating the ability to suppress oncogenic signaling both in vitro and in vivo [159]. In another approach, Ling et al. achieved direct capture of Ras protein and blockade of the ERK/AKT pathway by continuously expressing the Ra1 DNA aptamer intracellularly, which significantly inhibited the proliferation of lung cancer cells [160]. Furthermore, the applications of aptamers are continuously expanding. For instance, DeLong et al. loaded aptamers targeting the Ras‐binding domain onto ZnO nanoparticles, achieving targeted accumulation at tumor sites and Ras pathway imaging, thereby combining both drug delivery and therapeutic functions [161]. Meanwhile, Ma et al. also reviewed the platform‐based development prospects of aptamers targeting high‐value targets such as KRAS in the early detection and targeted therapy of gynecological tumors, highlighting their broad clinical application potential [162].

#### 3.1.6 mRNA Vaccines and piRNA

In nucleic acid‐based Ras‐targeting therapeutic strategies, mRNA vaccines and piRNA regulatory mechanisms represent emerging and promising research directions. mRNA vaccine technology offers innovative immunotherapy strategies for targeting Ras mutations. Preliminary clinical studies have validated its safety and feasibility. Rappaport et al. reported that the KRAS shared neoantigen vaccine combined with immune checkpoint inhibitors demonstrated good safety in a Phase I clinical trial and successfully induced TP53‐skewed T‐cell immune responses, laying a crucial foundation for subsequent efforts to optimize KRAS neoantigen vaccines [163]. Wang et al. further confirmed that an mRNA vaccine targeting the KRAS G12V mutation, when combined with the PD‐1 inhibitor pembrolizumab, produced clinical benefit in patients with advanced solid tumors, providing strong support for the concept of mutant KRAS as a viable immunotherapy target [111]. Cafri et al.’s study demonstrated that personalized mRNA vaccines could induce KRAS G12D‐specific T‐cell responses in patients with gastrointestinal cancer [110], and a Phase I trial of a multi‐epitope KRAS mutant long‐peptide vaccine with dual checkpoint blockade has further validated this immunotherapeutic approach in resected pancreatic cancer [164]. In terms of formulation innovation, Xu et al. designed a novel vaccine that co‐encapsulates the STING agonist cGAMP and KRAS G12D mRNA in LNPs. This strategy can reprogram the hepatic immune microenvironment, significantly inhibiting tumor metastasis and prolonging survival in pancreatic cancer mouse models [165]. In addition to the aforementioned strategies, piRNAs have also been found to be involved in the regulation of Ras‐related tumors. Research by Kim et al. revealed that Maelstrom, a key component of the piRNA processor, is abnormally elevated in various cancer cells. Loss of its function specifically induces cancer cell death by activating the ataxia telangiectasia mutated (ATM)‐mediated DNA damage‐apoptosis pathway and effectively blocks cell malignant transformation co‐driven by MYC and Ras [166]. Although the KRAS G12C inhibitors sotorasib and adagrasib have been approved, nucleic acid therapeutics offer complementary advantages, including broader mutation coverage, potential to overcome acquired resistance, and programmable design.

### 3.2 Myc

The MYC proto‐oncogene, as a core member of the MYC transcription factor family, is one of the most commonly dysregulated driver genes in human malignancies [167]. The MYC protein it encodes forms a heterodimer with the MYC‐associated factor X (MAX) protein, which specifically recognizes the E‐box *cis*‐acting element, thereby executing its core transcriptional regulatory function [168]. As a key integrator of internal cellular states and external proliferation signals, MYC exerts a decisive influence on cell proliferation, growth, and differentiation by orchestrating a series of core biological processes. These include cell cycle progression, ribosome biogenesis, cellular metabolism, protein synthesis, and telomerase activity through the global transcriptional regulation of a vast array of target genes [169, 170, 171]. In terms of oncogenic mechanisms, the activation of MYC is more commonly attributed to gene amplification, chromosomal translocations, or transcriptional dysregulation and protein overexpression resulting from aberrant upstream signaling pathways, rather than frequent mutations in the MYC gene itself [172]. This aberrant activation ultimately disrupts normal cellular homeostasis and drives the initiation and progression of over 70% of human malignancies. Its prevalence across various tumor types underscores its central role as a critical oncogene and therapeutic target [173].

#### 3.2.1 Mechanisms of “Undruggable”

**FIGURE 3:**
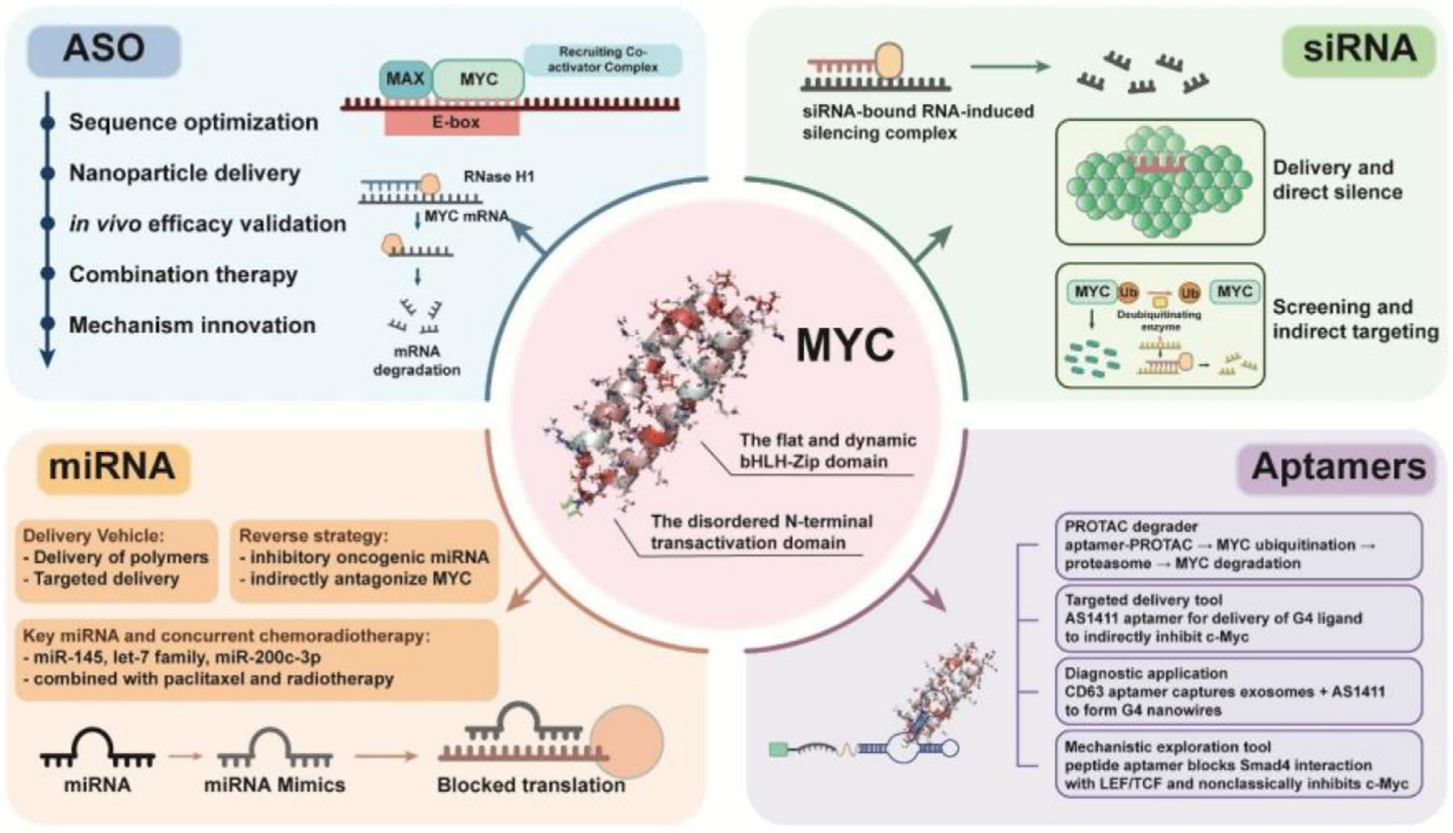
MYC Oncoprotein: A Multi‐Modal Arsenal of Nucleic Acid Strategies. This schematic systematically illustrates diverse nucleic acid modalities targeting MYC, including ASO‐ and siRNA‐mediated mRNA degradation, aptamer‐driven protein recognition, and miRNA replacement therapy for network regulation. The MYC protein structure image was generated with MolViz (https://molviz.com).

While the central role of MYC in tumorigenesis is well‐established, it has long been considered a prime example of an “undruggable” target. This is primarily due to the dual challenges posed by its inherent biochemical properties and physiological functions [174]. At the structural level, the MYC protein lacks the deep hydrophobic pockets typically found in classic drug targets like kinases, which are essential for high‐affinity small‐molecule binding [175]. Functionally, its transcriptional regulatory activity relies heavily on protein‐protein interactions and is highly context‐dependent, particularly involving the dynamic assembly of coactivator complexes and their collaborative interplay with other transcription factors [176]. At the functional level, the gene networks regulated by MYC are extensively involved in maintaining the physiological homeostasis of normal tissues, particularly those that are rapidly renewing. Therefore, systemic inhibition of MYC function may lead to severe on‐target toxicities, significantly restricting the therapeutic window of potential treatment strategies [5]. Despite these formidable challenges, the research field is actively pursuing breakthroughs through innovative strategies. These include targeting its transcriptional co‐factors, utilizing protein degradation technologies to directly degrade the MYC protein, and applying ASOs to inhibit its expression. These cutting‐edge explorations offer new hope for ultimately conquering this “undruggable” target [174, 177] (Figure 3).

#### 3.2.2 ASOs

c‐Myc, a key member of the MYC family of oncogenic transcription factors, presents an “undruggable” nature, making mRNA‐targeting ASO strategies a highly attractive therapeutic direction. Exploration in this field has progressed from proof‐of‐concept to optimization stages focused on delivery efficiency and combination therapies. Early studies laid the groundwork for the feasibility of ASOs targeting MYC. Research by Yu et al. first validated the dependency on the c‐Myc oncogene, demonstrating that phosphorothioate‐modified anti‐c‐Myc ASOs successfully inhibited proliferation and promoted differentiation of human colon cancer cell lines in vitro. This indicates that directly targeting c‐Myc mRNA is an effective anticancer strategy [178]. Subsequently, through sequence optimization, Ye et al. designed a novel c‐Myc ASO3, demonstrating in A549 lung cancer cells its ability to effectively downregulate both c‐Myc mRNA and protein levels and efficiently induce apoptosis, providing an excellent candidate drug sequence for further development [179]. Based on the demonstration of its in vitro efficacy, research has progressively shifted toward overcoming barriers in solid tumor therapy, with a particular focus on addressing delivery challenges. Oliveira et al. employed gold nanoparticles as carriers to deliver anti‐c‐Myc ASOs, successfully achieving gene silencing effects comparable to those in 2D cultures within more clinically relevant 3D tumor spheroid models. This provides a crucial dosimetric basis for addressing the effective delivery of ASOs in complex tumor microenvironments [180]. The in vivo study by Dhanasekaran et al. has significantly advanced the translational prospects of this field. In transgenic mouse models of MYC‐driven liver and kidney cancers, they demonstrated that MYC‐ASOs not only effectively inhibited tumor growth and induced an “oncogene addiction” effect but also exhibited low toxicity. This provides robust preclinical evidence for the application of MYC‐ASO therapy in various solid tumors [181]. In addition to directly targeting MYC itself, ASO strategies aimed at its upstream regulatory molecules also demonstrate significant potential, particularly in the context of combination therapies. Yang et al. discovered that in KRAS‐mutant non‐small cell lung cancer, the long non‐coding RNA HIF1A‐As2 forms a positive feedback loop with MYC. Using ASOs to block HIF1A‐As2 significantly enhanced the efficacy of MYC inhibitors combined with cisplatin, thereby offering a novel perspective for disrupting the MYC regulatory network indirectly to overcome drug resistance [182]. Furthermore, ASO technology itself is undergoing continuous innovation. Moving beyond the conventional inhibitory mechanisms of traditional ASOs, Liu et al. employed chemical evolution to develop a DNAzyme with ASO‐like properties. This novel construct is capable of precisely cleaving c‐Myc mRNA within colon cancer cells, subsequently inducing cell cycle arrest and apoptosis. Their work establishes a groundbreaking new platform for anticancer nucleic acid therapeutics [183]. In summary, the therapeutic landscape of MYC‐targeting ASOs has evolved from early in vitro validation to current assessments of in vivo efficacy, the development of novel delivery systems, and the exploration of combination strategies. This progression has revealed a diversified development paradigm shifting from monotherapy to combination approaches and from traditional mechanisms to innovative platforms. These advances offer a promising translational pathway toward addressing this historically “undruggable” target.

#### 3.2.3 siRNA

As a pivotal oncogenic driver, c‐Myc represents a promising therapeutic target. A highly attractive strategy is the direct silencing of c‐Myc expression using siRNA technology. Alternatively, intervening in its upstream regulatory network via siRNA also holds significant therapeutic potential. In the context of direct targeting, researchers have focused on developing efficient delivery systems to achieve effective c‐Myc gene silencing. For instance, Anilmis NM and colleagues successfully engineered plant oil‐based nanoparticles for the delivery of c‐Myc siRNA and demonstrated their gene‐silencing efficacy and anti‐cancer activity in lung cancer cells, offering a novel biocompatible tool for siRNA delivery [184]. Likewise, Huang W and colleagues developed a targeted nanosystem capable of co‐delivering doxorubicin and c‐Myc siRNA. This system achieved synergistic enhancement of chemotherapy and gene therapy in a glioblastoma model, while also demonstrating favorable tumor penetrability [185]. The review by Padayachee J and colleagues systematically summarizes strategies utilizing non‐viral nanocarriers for delivering siRNA to silence oncogenes such as MYC, providing an in‐depth analysis of their mechanisms and clinical translational potential in breast cancer therapy [186]. In addition to directly silencing MYC itself, another critical research direction involves utilizing siRNA for functional screening to uncover its upstream regulatory factors, thereby facilitating the development of indirect targeting strategies. Numerous studies have employed large‐scale siRNA screening to identify key deubiquitinating enzymes that regulate c‐Myc protein stability and thereby influence its function. For instance, Li M et al. demonstrated that USP43 stabilizes c‐Myc through deubiquitination, thereby promoting glycolysis and metastasis in bladder cancer [187]. Jiang L et al. further demonstrated that knockdown of WDR20 induces senescence in hepatocellular carcinoma cells by impairing the deubiquitination of c‐Myc mediated by the USP12/46 complex [188]. Meanwhile, screening conducted by Tu R et al. revealed that USP29 concurrently deubiquitinates and stabilizes both MYC and HIF1α, thereby cooperatively driving tumor metabolism and progression [189]. These collective findings indicate that targeting these deubiquitinating enzymes represents a viable novel strategy for indirectly modulating the stability of c‐Myc protein.

Furthermore, siRNA screening has elucidated alternative pathways for the indirect modulation of c‐Myc. For instance, Yamamoto V and colleagues demonstrated that siRNA‐mediated knockdown of the endoplasmic reticulum stress chaperone GRP78 upregulates the translational repressor 4E‐BP1, thereby inhibiting the synthesis of c‐Myc at the translational level [190]. Chen X et al. further confirmed through siRNA knockdown that hnRNP H and F proteins regulate a MYC‐dependent alternative splicing event of HRAS, thereby influencing the fate of prostate cancer cells. This finding unveils an alternative regulatory mechanism of MYC at the level of RNA splicing [191]. To systematically identify such regulatory factors, Kallal LA and colleagues developed a high‐throughput siRNA screening and validation platform centered on c‐Myc protein levels as the primary readout, providing a powerful tool for the rapid identification of small molecules capable of indirectly modulating c‐Myc [192]. Indeed, MYC‐targeting siRNA strategies have demonstrated distinct value in remodeling the tumor microenvironment. Research by Yan X et al. revealed that nanoparticle‐mediated delivery of siRNA targeting MYC in tumor vascular endothelial cells mimics the effects of Notch activation, promoting the normalization of aberrant vasculature. This enhances the efficacy of both chemotherapy and immunotherapy, offering a novel combinatorial therapeutic approach for siRNA therapy that extends beyond direct tumor cell cytotoxicity [193]. In summary, siRNA‐based strategies provide a diversified arsenal to address the c‐Myc challenge. On the one hand, advanced nano‐delivery systems enable direct and efficient gene silencing or combination therapy. On the other hand, functional screening continuously unveils c‐Myc's intricate regulatory network, identifying numerous upstream targets that indirectly modulate its stability, translation, and function. This demonstrates substantial potential for integration with existing treatments.

#### 3.2.4 miRNA

In addition to ASOs and siRNA, the use of miRNA replacement therapy to restore the function of endogenous MYC suppressors represents another highly promising nucleic acid‐based therapeutic strategy. Extensive studies have demonstrated that multiple miRNAs downregulated in tumors directly target MYC mRNA. Restoring the expression of these miRNAs effectively inhibits MYC‐driven tumor progression and exhibits synergistic potential with conventional therapies. Numerous investigations have systematically identified key miRNAs that directly target MYC across various cancer types. In their systematic review, Shams et al. identified six miRNAs, including let‐7a and miR‐145, which impede the progression of pancreatic ductal adenocarcinoma by directly suppressing MYC expression. These findings provide well‐characterized miRNA candidates for replacement therapy against this challenging MYC‐driven malignancy [194]. Similarly, Pourteimoor et al. highlighted that miRNAs such as miR‐342 and miR‐520 can achieve subtype‐specific inhibition by downregulating c‐Myc in breast cancer, and further proposed novel prognostic tools based on exosomal or circulating miRNAs associated with c‐Myc [195].

Among candidate miRNAs, miR‐145 stands out for its potent MYC suppression. Ibrahim et al. demonstrated that PEI‐mediated delivery of unmodified miR‐145 suppressed tumor growth in a colon cancer mouse model, accompanied by c‐Myc downregulation and enhanced apoptosis, thereby providing proof‐of‐concept for direct miRNA replacement therapy [55]. To improve delivery, Liang et al. designed a hyaluronic acid‐modified nanocarrier that efficiently targets miR‐145 to colon tumors, inducing cell cycle arrest, apoptosis, and tumor suppression via c‐Myc downregulation [196]. Other miRNAs, including the let‑7 family and miR‑200c‑3p, show promise in countering therapy resistance. Nadiminty et al. found that let‑7c suppresses c‐Myc, reducing androgen receptor expression and proliferation in prostate cancer, suggesting its restoration may overcome castration resistance [197]. Anastasiadou et al. reported that miR‑200c‑3p downregulates β‑catenin and c‑Myc in ovarian cancer and reverses chemoradiation‑induced PD‑L1 upregulation, synergizing with standard therapies to inhibit growth and overcome resistance [198].

The synergistic effects of miRNA replacement therapy with chemotherapy and radiotherapy have been well established. For instance, Hejazi et al. demonstrated that combining miR‑193a with paclitaxel suppresses migration and clonogenicity in colon cancer by inhibiting c‑Myc and related metastatic pathways [199]. Similarly, Mousavikia et al. reported that miR‑16‑5p enhances radiosensitivity in colon cancer through targeting the PI3K/AKT/mTOR pathway and downregulating MYC [200]. Notably, modulating the miRNA network itself offers an indirect strategy for MYC regulation. In a distinct approach, Dhanasekaran et al. used LNPs to deliver anti‑miR‑17. This silences the oncogenic miRNA and relieves its repression on target genes, such as pro‑apoptotic factors downstream of MYC, thereby inducing apoptosis and delaying tumorigenesis in MYC‑driven liver cancer [201]. In summary, tumor‑suppressive miRNAs such as miR‑145 and let‑7 form an endogenous network that finely tunes MYC expression. Restoring their function via advanced delivery systems not only directly inhibits MYC but also synergizes with chemotherapy, radiotherapy, endocrine therapy, and immunomodulation across cancer types, offering a promising combinatorial strategy to counteract MYC‑driven malignancy and therapy resistance.

#### 3.2.5 Aptamers

In addition to miRNA replacement therapy, aptamers represent another precise targeting strategy against c‐Myc. Building upon these highly specific aptamers, the most advanced progress involves converting them into proteolysis‐targeting chimeras (PROTACs), enabling the complete elimination of c‐Myc. For instance, based on the selected aptamer MA9C1, Wang et al. innovatively engineered a PROTAC molecule named ProMyc. This molecule effectively recruits intracellular E3 ubiquitin ligases to ubiquitinate c‐Myc, leading to its degradation via the proteasome pathway, which resulted in significant tumor regression in animal models and provided a novel strategy for directly degrading c‐Myc [72]. Furthermore, leveraging a bivalent Threose Nucleic Acid (TNA) aptamer that binds the c‐Myc/Max complex, Li et al. developed a TEP PROTAC. This molecule not only effectively degrades the c‐Myc/Max complex but also demonstrates synergistic effects with the CDK4/6 inhibitor palbociclib, collectively suppressing the growth of triple‐negative breast cancer [202].

Beyond directly targeting the c‐Myc protein, aptamers also play significant roles in other related fields. For instance, Carvalho et al. utilized the well‐known nucleolin‐targeting aptamer AS1411 in combination with the G‑quadruplex ligand C8 to achieve selective delivery to cervical cancer cells, successfully suppressing c‑MYC expression while reducing toxicity to normal cells [203]. Zheng et al. cleverly integrated the CD63 aptamer with exosome capture technology and employed AS1411 to form G‑quadruplex nanowires for amplifying fluorescence signals, enabling highly sensitive detection of tumor‑derived exosomes and offering a novel tool for cancer diagnosis [204]. Additionally, aptamers have been applied to explore novel regulatory mechanisms of c‑Myc. Lim et al. designed a peptide aptamer that specifically blocks the Smad4‐LEF/TCF interaction, thereby inhibiting c‑Myc expression and slowing hepatocellular carcinoma cell growth without TGF‑β signaling. This finding reveals a non‑canonical pro‑proliferative function of Smad4 [205]. In a different approach, Morrissey et al. employed G‑quadruplex sequences derived from the promoter regions of genes such as c‐Myc as “decoys” to successfully capture and identify new potential aptamer targets, including nucleolin and RPL19, thereby broadening the sources for aptamer discovery [206]. In summary, aptamers, owing to their high specificity and ease of engineering, have emerged as a unique and versatile tool for targeting c‐Myc. Their applications extend beyond traditional competitive inhibition to advanced strategies such as PROTAC‐mediated protein degradation, while also demonstrating considerable potential in tumor‑targeted delivery, mechanistic exploration, and highly sensitive diagnostics. Together, they constitute a diversified technological platform encompassing therapy, diagnosis, direct intervention, and mechanistic investigation, as systematically reviewed in a 2026 framework paper on MYC‐reducing strategies including aptamer‐based PROTACs [207].

### 3.3 p53

p53 is a critical tumor suppressor protein situated at the nodal point of multiple cellular stress signaling pathways. As a transcription factor, it regulates key biological processes, including the cell cycle, DNA repair, senescence, and apoptosis, thereby playing an essential role in maintaining genomic stability [4, 208]. In human cancers, TP53 is the most frequently mutated gene, and its functional inactivation is a common event in tumorigenesis [209].

#### 3.3.1 Mechanisms of “Undruggable”

**FIGURE 4:**
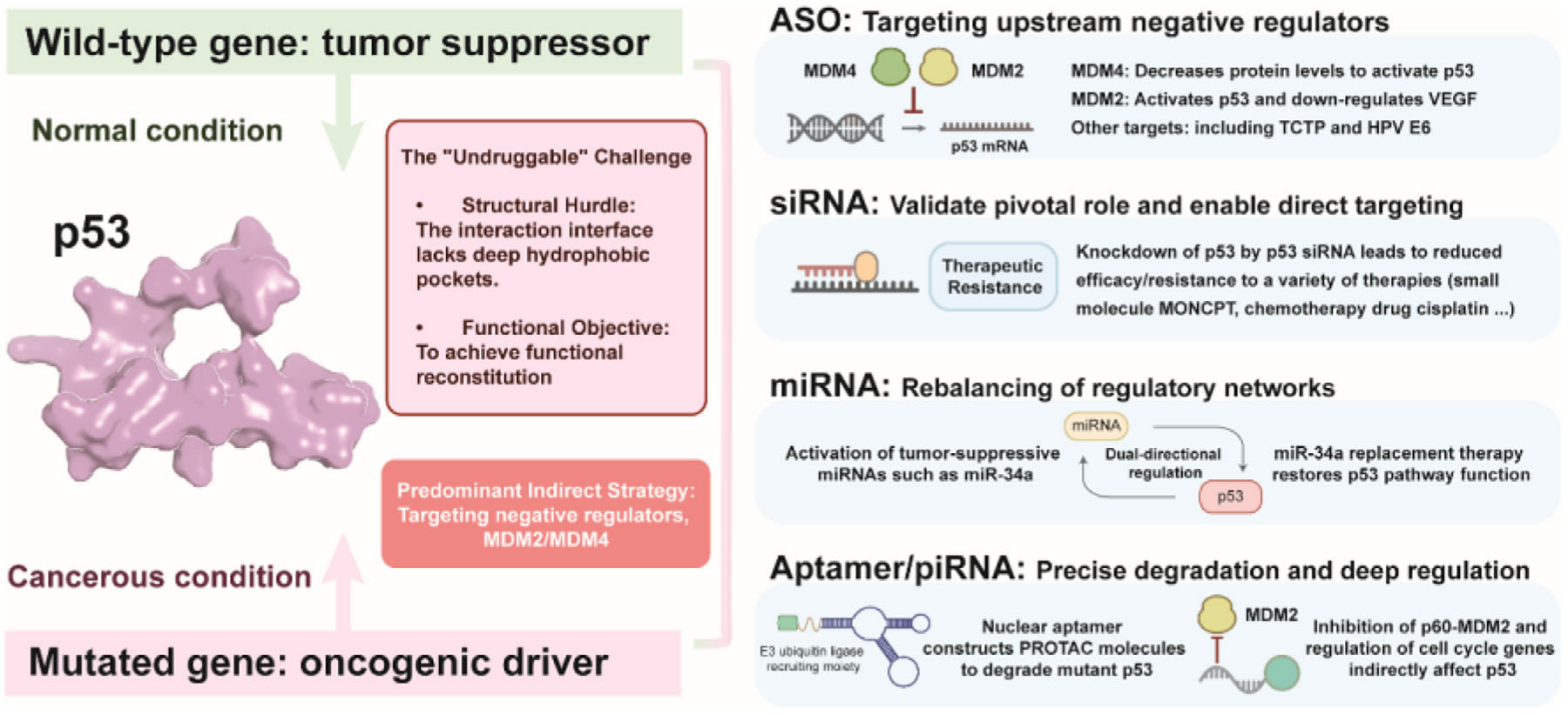
p53 conundrum: nucleic acid strategies for reconstitution and degradation. This schematic summarizes two principal nucleic acid intervention strategies for p53, including the restoration of wild‐type function by targeting its negative regulators, and the specific degradation of oncogenic mutants via aptamer‐based PROTACs. MDM2/4: Mouse Double Minute 2/4; TCTP: Translationally Controlled Tumor Protein; HPV16 E6: Human Papillomavirus Type 16 Early Protein 6; MONCPT: 7‐((4‐Methylpiperazin‐1‐yl) methyl)‐10,11‐methylenedioxy‐20(S)‐camptothecin. The p53 protein structure image was generated with MolViz (https://molviz.com).

Nevertheless, p53 is widely recognized as an “undruggable” target [210]. This perception stems primarily from two fundamental challenges. First, the core function of p53 depends on its properties as a transcription factor. Its protein surface lacks deep hydrophobic pockets suitable for conventional small‐molecule drug binding, and specifically modulating its extensive, flat interaction interfaces with DNA or co‐activators presents substantial technical difficulties. Second, the majority of p53 mutations are loss‐of‐function mutations. The goal of drug development is therefore not to inhibit abnormal activity, but to restore or mimic the complex transcriptional functions of the wild‐type protein, a strategy of functional reconstitution that poses significant pharmacological hurdles [211, 212]. Current drug development efforts have consequently focused largely on indirect approaches, such as antagonists targeting its negative regulator mouse double minute (MDM) [213], with recent reviews further delineating how specific p53 mutations dictate differential responses to conventional therapies [214] (Figure 4).

#### 3.3.2 ASOs

ASOs technology offers a precise strategy to restore key tumor suppressor pathways through indirect mechanisms. One important therapeutic approach involves using ASOs to target negative regulators of the tumor suppressor p53, thereby reactivating its function. This strategy encompasses several mechanisms: First, ASOs can directly target intrinsic cellular repressors of p53, such as MDM2 and MDM4. For example, Dewaele et al. designed an ASO that induces skipping of exon 6 in MDM4 pre‑mRNA, effectively reducing MDM4 protein levels, relieving p53 inhibition, and suppressing tumor growth while enhancing sensitivity to targeted therapy in melanoma and lymphoma models [215]. Similarly, work by Xiong et al. in breast cancer models demonstrated that MDM2‐targeted ASOs induce p53 activation and elicit a concomitant, mechanistically dual downregulation of VEGF, orchestrating an anti‐angiogenic response not achievable with the small‐molecule inhibitor nutlin‐3 [216]. Second, ASOs can be directed against other oncoproteins that indirectly modulate p53. Baylot et al. reported that in castration‑resistant prostate cancer, an ASO targeting the translationally controlled tumor protein (TCTP) restores p53 expression and function, and shows synergistic potential when combined with castration or chemotherapy [217]. Moreover, targeting viral oncogenes represents another application. Reschner et al. developed an innovative ruthenium‑labeled ASO that specifically binds to the HPV16 E6 oncogene. Upon photoactivation, it irreversibly crosslinks and disrupts the target sequence, suppresses E6 protein expression, reactivates p53, and effectively inhibits the proliferation of cervical cancer cells [218]. In summary, these studies collectively demonstrate that ASOs can target upstream negative regulators of the p53 pathway, such as MDM2/MDM4, TCTP, and HPV E6. This provides an effective strategy to indirectly restore p53 tumor suppressor function and inhibit tumor growth, thereby expanding the arsenal of cancer therapeutics.

#### 3.3.3 siRNA

The tumor suppressor p53 plays a central role in mediating cellular responses to various anticancer therapies, and its functional status directly influences treatment efficacy. Studies have shown that knockdown of p53 expression using p53 siRNA significantly diminishes the effectiveness of multiple therapies with distinct mechanisms of action. For example, Zhang et al. found that the novel anticancer compound MONCPT induces G2/M cell cycle arrest in lung cancer A549 cells through the p53 and p38 MAPK pathways; however, when p53 function was disrupted with p53 siRNA, this cell cycle arrest was markedly delayed [219]. Regarding chemotherapeutic agents, Köberle et al. demonstrated that the robust apoptotic response of testicular tumor cells to cisplatin depends on the p53 and Fas pathways. Knocking down p53 suppressed the death receptor‑mediated apoptotic pathway, thereby increasing resistance to cisplatin [220]. Similarly, p53 acts as an indispensable mediator in combination therapies. Gong et al. reported that the combined apoptotic effect of vitamin C and the non‑steroidal anti‑inflammatory drug sulindac in colon cancer is mediated by p53. They further showed that siRNA‑mediated knockdown of p53 induced cellular resistance to this combination therapy [221]. Another study by Braicu et al. revealed in triple‑negative breast cancer that co‑treatment with the phytochemical epigallocatechin gallate and p53 siRNA unveiled a synergistic effect in activating pro‑apoptotic genes and suppressing pro‑survival genes, further underscoring the importance of the p53 pathway in this synergy [222]. Additionally, directly targeting p53 itself may exert anticancer effects. For instance, Braicu et al. observed in a cervical cancer model that p53 siRNA alone reduced cell migration and downregulated genes associated with apoptosis and angiogenesis [223]. In summary, these studies collectively highlight that an intact p53 pathway is essential for maintaining the efficacy of diverse anticancer therapies, such as small‑molecule compounds, chemotherapeutic drugs, and natural products, and that loss of p53 function represents a key mechanism underlying treatment resistance.

#### 3.3.4 miRNA

The p53 tumor suppressor pathway and miRNAs form an extensive and intricate bidirectional regulatory network, which plays a central role in tumor suppression and cancer therapy. On the one hand, as a transcription factor, p53 directly regulates the expression of several key miRNAs, most notably miR‑34a. Li et al. elaborated on miR‑34a as a pivotal tumor suppressor capable of effectively inhibiting cancer stem cells and positively activating the p53 pathway, highlighting its potential as a novel anticancer therapeutic agent [224]. Fawzy et al. further emphasized in a systematic review that restoring p53 pathway function through nanocarrier‑mediated delivery of miR‑34a has become an important strategy for enhancing the efficacy of various cancer immunotherapies [225]. Beyond miR‑34a, other miRNAs also participate in the fine‑tuning of the p53 pathway. For instance, Huang et al. demonstrated that miR‑340 can directly target MDM2 to stabilize p53 protein, thereby suppressing prostate cancer proliferation and metastasis [226].

On the other hand, the functional status of p53 profoundly influences the global miRNA expression profile. When p53 is mutated, it not only loses its tumor‑suppressive functions but may also acquire novel oncogenic properties, a phenomenon termed gain‑of‑function (GOF). One such GOF mechanism involves disrupting miRNA biogenesis. Gurtner et al. investigated how mutant p53 interferes with the Drosha complex, leading to global dysregulation of miRNA biogenesis, a finding that offers fresh insights into tumorigenesis and therapy resistance [227]. This dysregulation holds significant clinical implications. For example, Eichelmann et al. revealed that mutant p53 modulates drug sensitivity in esophageal adenocarcinoma by affecting specific miRNAs, including miR‑27a‑5p and miR‑30e‑5p, and their downstream target SLC7A11, demonstrating prognostic and predictive value for treatment response [228].

This intricate regulatory network functions as a robust tumor‑suppressive hub, whose dysregulation represents a hallmark feature of cancer. Reviews by Sargolzaei et al. and Singh et al. have systematically outlined the role of the p53‑miRNA regulatory network as a tumor‑suppressive hub and highlighted that targeting this circuitry offers novel potential avenues for therapeutic intervention in cancer [229, 230]. Building on this understanding, new treatment strategies are emerging. For instance, Hong et al. demonstrated that a traditional Chinese medicine compound can downregulate the oncogenic miRNA‑21‑3p, thereby relieving its suppression of the p53 pathway and enhancing the chemotherapeutic efficacy of 5‑fluorouracil in gastric cancer [231]. Similarly, through bioinformatic analysis and experimental validation, Xie et al. proposed that miRNA‑302s may regulate ARL4C‑driven gastric cancer progression via the p53 signaling pathway, suggesting a new potential therapeutic target [232]. In summary, p53 and miRNAs engage in a dynamic, bidirectional dialogue network. A deeper understanding of this network not only unveils novel mechanisms underlying tumor initiation and progression, but also opens promising avenues for developing innovative anticancer strategies, such as miR‑34a replacement therapy, and for overcoming therapy resistance.

#### 3.3.5 Aptamers

With the continuous advancement of nucleic acid drug technology, novel molecular tools such as aptamers offer unprecedented strategies for precise intervention in the p53 pathway. Regarding aptamers, their applications have evolved from simple binding and inhibition to the construction of complex PROTACs. For example, Kong et al. rationally engineered a DNA aptamer that directly and with high affinity binds to the p53‐R175H hotspot mutant. Using this aptamer as a core component, they developed a PROTAC‐based degrader capable of specifically inducing the degradation of mutant p53, providing a novel and precise therapeutic strategy for eliminating oncogenic mutant p53 proteins [73]. In a complementary approach, Huang et al. designed and validated an RNA aptamer that specifically binds to wild‑type p53 protein. This aptamer was further engineered into a “ribozyme switch” that senses the activity status of intracellular p53 and regulates the output of downstream therapeutic gene circuits, achieving precise tumor cell elimination [233]. Additionally, aptamers can be utilized in targeted delivery systems to indirectly influence p53. For instance, Zhou et al. constructed a targeted microemulsion using the SYL3C aptamer, which directly recognizes and binds to surface targets on cancer cells. This system not only enables targeted delivery but also downregulates the expression of downstream mutant p53 protein, which represents a key mechanism underlying its anticancer effects [234].

#### 3.3.6 piRNA

In addition to aptamers, piRNAs, a class of small non‑coding RNAs, have also been found to be deeply involved in the regulation of the p53 pathway. For instance, Wang et al. discovered that in lung squamous cell carcinoma, piR‑L‑138 directly binds to and inhibits the function of p60‑MDM2, a key negative regulator of p53, thereby impairing p53‑mediated apoptosis and ultimately inducing resistance to cisplatin chemotherapy. This finding provides a novel intervention target for overcoming drug resistance [90]. On the other hand, Zhang et al. reported that Nuclear RNA Export Factor 3 regulates gastric cancer progression through piRNA‑related pathways. Specifically, piRNA‐3457319 is involved in modulating the p53 signaling pathway by targeting cell‑cycle‑related genes such as Cyclin D1 and CDK Inhibitor 1A, revealing a novel mechanism of the piRNA‑p53 network in cancer [235]. In summary, the precision degradation and sensing technologies based on aptamers, along with the deep regulatory roles of piRNAs in the p53 pathway, collectively represent cutting‑edge explorations in nucleic‑acid‑based drug development targeting p53 as a central tumor‑suppressive hub. These strategies not only deepen our understanding of the complexity of the p53 regulatory network but also open promising new directions for developing specific therapies tailored to different p53 statuses, such as activating wild‑type p53 or degrading mutant forms.

## 4 Overcoming the Core Mechanism of “undruggable”

**FIGURE 5:**
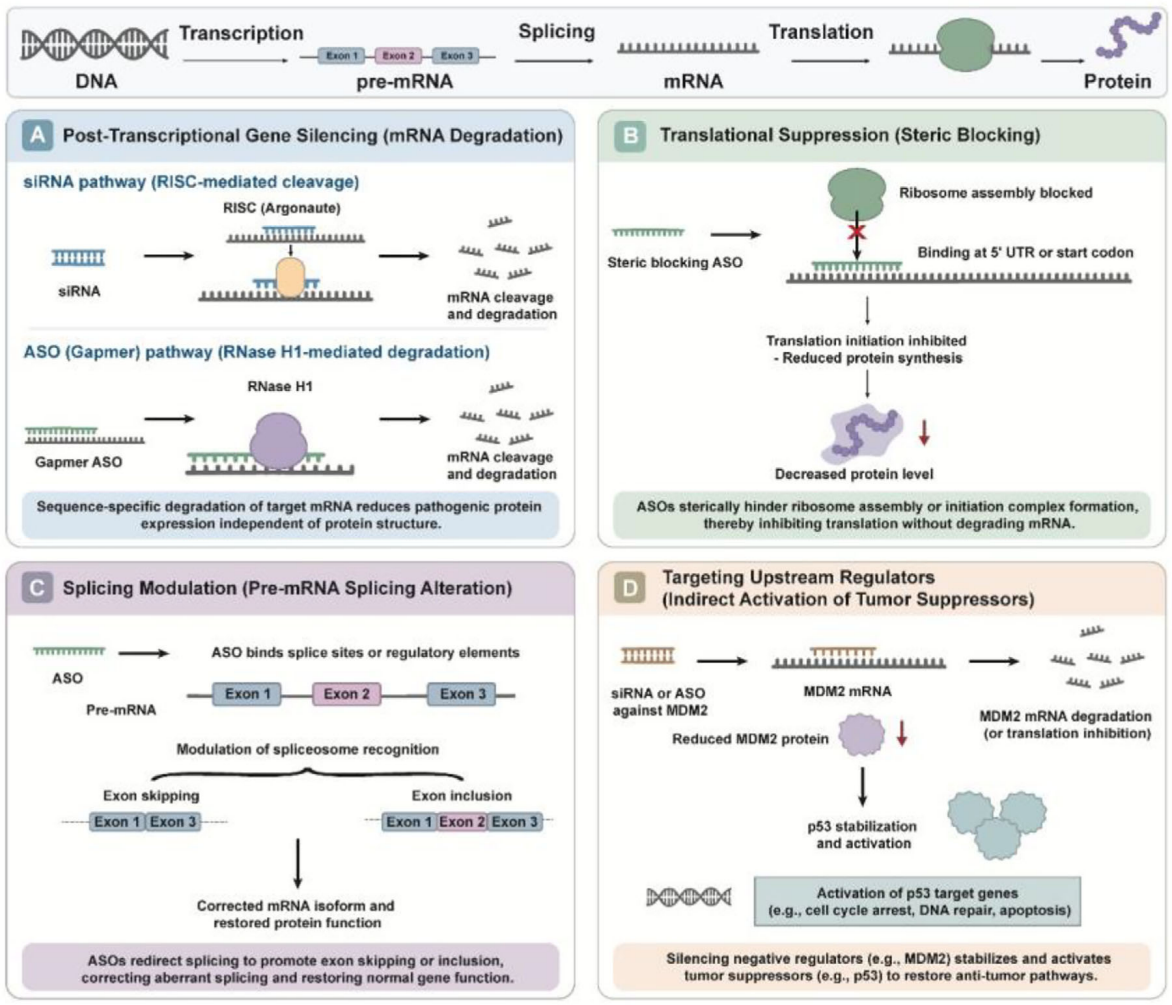
Schematic illustration of nucleic acid therapeutic mechanisms for overcoming undruggable targets. This diagram summarizes four intervention strategies. Panel A depicts post‐transcriptional gene silencing, where antisense oligonucleotides (ASOs) recruit RNase H1 or small interfering RNAs (siRNAs) load into the RNA induced silencing complex (RISC) to degrade target mRNA. Panel B shows translational suppression, where steric blocking ASOs prevent ribosome assembly and translation initiation. Panel C presents splicing modulation, where ASOs bind to splice sites or regulatory elements in pre‐mRNA to alter exon inclusion patterns. Panel D demonstrates targeting of upstream regulators, where siRNAs or ASOs against MDM2 relieve p53 inhibition and restore tumor suppressor function. This mechanistic overview highlights how nucleic acid therapeutics circumvent the need for direct protein binding by operating at the mRNA level.

Traditional drug discovery relies on high‐affinity binding to functional domains of proteins. However, this approach presents a significant hurdle for targets lacking such binding pockets or those involving extensive protein‐protein interactions. Nucleic acid therapeutics circumvent this fundamental limitation by intervening at the upstream level of the central dogma, directly modulating the flow of genetic information (Figure 5), and thus offering a disruptive strategy to target these previously “undruggable” molecules [236].

### 4.1 Post‐Transcriptional Gene Silencing

Post‐transcriptional gene silencing (PTGS) is currently the most established nucleic acid strategy for targeting “undruggable” proteins. It achieves therapeutic goals by directly degrading the mRNA template that encodes the pathogenic protein [237, 238]. siRNA, upon incorporation into the RISC, directs the sequence‐specific degradation of target mRNA via complementary base‐pairing [239]. This mechanism is independent of the 3D structure of the target protein and requires only recognition of a specific mRNA sequence, making it particularly advantageous for downregulating oncogenes such as mutant Ras [240]. Studies have shown that siRNAs designed against the KRAS G12D mutation can selectively eliminate the oncogenic transcript while preserving the integrity of wild‐type gene function, demonstrating excellent selectivity [241, 242]. With continuous improvements in delivery systems, siRNA therapies based on LNPs or GalNAc conjugation technology are now under clinical evaluation [243, 244]. Similarly, Gapmer‐type ASOs hybridize to target mRNAs via their DNA core region and recruit endogenous RNase H1 to degrade the mRNA [245]. This mechanism also avoids direct interaction with the target protein and has shown significant efficacy in targeting MYC. Experimental evidence confirms that MYC‐specific ASOs effectively reduce MYC protein levels and suppress tumor cell proliferation, providing key proof of concept for regulating transcription factors traditionally considered “undruggable” [181].

### 4.2 Translational Suppression

Apart from direct degradation, certain heavily chemically modified ASOs function via steric hindrance. These molecules do not activate RNase H1 but instead bind with high affinity to translation initiation sites or ribosomal binding regions on the mRNA, physically blocking the assembly or progression of the translation initiation complex, thereby inhibiting protein synthesis [15, 246]. This approach is suitable for scenarios requiring fine‐tuned modulation rather than complete protein knockout.

### 4.3 Splicing Modulation

For diseases driven by aberrant splicing, ASOs can act as “molecular scalpels”, precisely correcting the erroneous flow of genetic information. By specifically binding to splicing regulatory elements in pre‐mRNA, ASOs can promote the skipping or inclusion of specific exons, thereby correcting pathogenic splicing events and generating functional mature mRNA [247]. Although this technique has limited direct application in targeting Ras/MYC/p53, its success in non‐oncological diseases demonstrates the versatility of nucleic acid therapeutics in addressing dysfunctional gene regulation and provides a paradigm for targeting other driver genes reliant on aberrant splicing [248].

### 4.4 Targeting Upstream Regulators

For loss‐of‐function targets such as p53, nucleic acid therapeutics can achieve indirect intervention by targeting their upstream negative regulators [249]. MDM2, a key negative regulator of p53, can be downregulated by siRNA or ASOs, effectively stabilizing p53 protein levels and reactivating the tumor suppressor pathway [250]. This strategy transforms the challenge of directly targeting p53 into the more tractable task of modulating MDM2.

## 5 Challenges and Strategies for Resolution

Currently, most relevant candidate drugs remain in the preclinical or early‐stage clinical research phase (Table 2). However, the efficacy of these therapies still requires validation through larger‐scale clinical trials. Meanwhile, emerging technologies such as aptamer based PROTACs are still primarily at the stage of preclinical model validation, and their translation into clinical applications faces additional unknown challenges.

**TABLE 2:** Nucleic acid drugs against “undruggable” targets in clinical translation.

INTERVENTION STRATEGY	TARGET MOLECULE	DRUG/MOLECULE TYPE	KEY FINDINGS	REF.
ASOs	c‐Myc	MYC‐ASO	Effectively inhibited tumor growth and induced an “oncogene addiction” effect with low toxicity in transgenic mouse models of MYC‐driven liver and kidney cancer.	[181]
	MDM4	MDM4 exon‐skipping ASO	Mediated skipping of exon 6 in MDM4 pre‐mRNA, reducing protein levels, relieving p53 inhibition, and inhibiting tumor growth while enhancing sensitivity to targeted therapy in melanoma and lymphoma models.	[215]
	MDM2	MDM2‐targeting ASO	Activated p53 and specifically downregulated VEGF expression to inhibit angiogenesis in a breast cancer model.	[216]
	TCTP	TCTP‐targeting ASO	Restored p53 expression and function in castration‐resistant prostate cancer, showing synergistic potential with castration or chemotherapy.	[217]
	HPV16 E6	Ruthenium‐labeled photoactivatable ASO	Targeted the HPV16 E6 oncogene. Light activation caused irreversible destruction of the target sequence, inhibiting E6 protein expression, reactivating p53, and suppressing cervical cancer cell proliferation.	[218]
	HRAS	ISIS 2503	A Phase I clinical trial demonstrated good safety and induction of disease stabilization in patients with advanced solid tumors, providing the first clinical validation of Ras inhibition feasibility.	[23]
	KRAS	AZD4785	Specifically reduced KRAS mRNA and protein levels, inhibited the MAPK pathway, and significantly shrunk tumors in xenograft models. Currently in preclinical development.	[24]
mRNA VACCINES	KRAS	Personalized mRNA vaccine	Induced KRAS G12D‐specific T‐cell responses in patients with gastrointestinal cancers, providing a rationale for combination immunotherapy.	[110]
	KRAS	mRNA vaccine	Combined with pembrolizumab in patients with advanced solid tumors, resulting in clinical benefit, supporting mutant KRAS as a viable immunotherapeutic target.	[111]
	KRAS	cGAMP/KRAS G12D mRNA LNP vaccine	Reprogrammed the liver immune microenvironment, significantly inhibiting tumor metastasis and prolonging survival in a pancreatic cancer mouse model.	[165]
	Shared KRAS neoantigens	KRAS shared neoantigen vaccine	A Phase I trial showed good safety and successfully induced a T‐cell immune response biased toward TP53.	[163]
APTAMERS	c‐Myc	ProMyc	Recruited E3 ubiquitin ligase to ubiquitinate and degrade c‐Myc, leading to significant tumor regression in animal models.	[72]
	p53‐R175H	dp53m	Specifically induced degradation of the oncogenic mutant p53 protein (R175H).	[73]

### 5.1 Delivery System Problems

The clinical translation of nucleic acid therapeutics for cancer treatment faces several delivery problems. First, these agents are susceptible to degradation by nucleases in the bloodstream. Second, the dense extracellular matrix and high interstitial pressure of solid tumors hinder deep penetration into tumor tissues. Third, inefficient endosomal escape after cellular uptake limits cytoplasmic delivery [251]. To address these problems, various strategies are being developed, including ligand‐receptor targeting approaches [243, 252], lipid nanoparticle optimization [253, 254], and exosome‐based carriers [255, 256, 257, 258].

### 5.2 Safety Concerns

**FIGURE 6:**
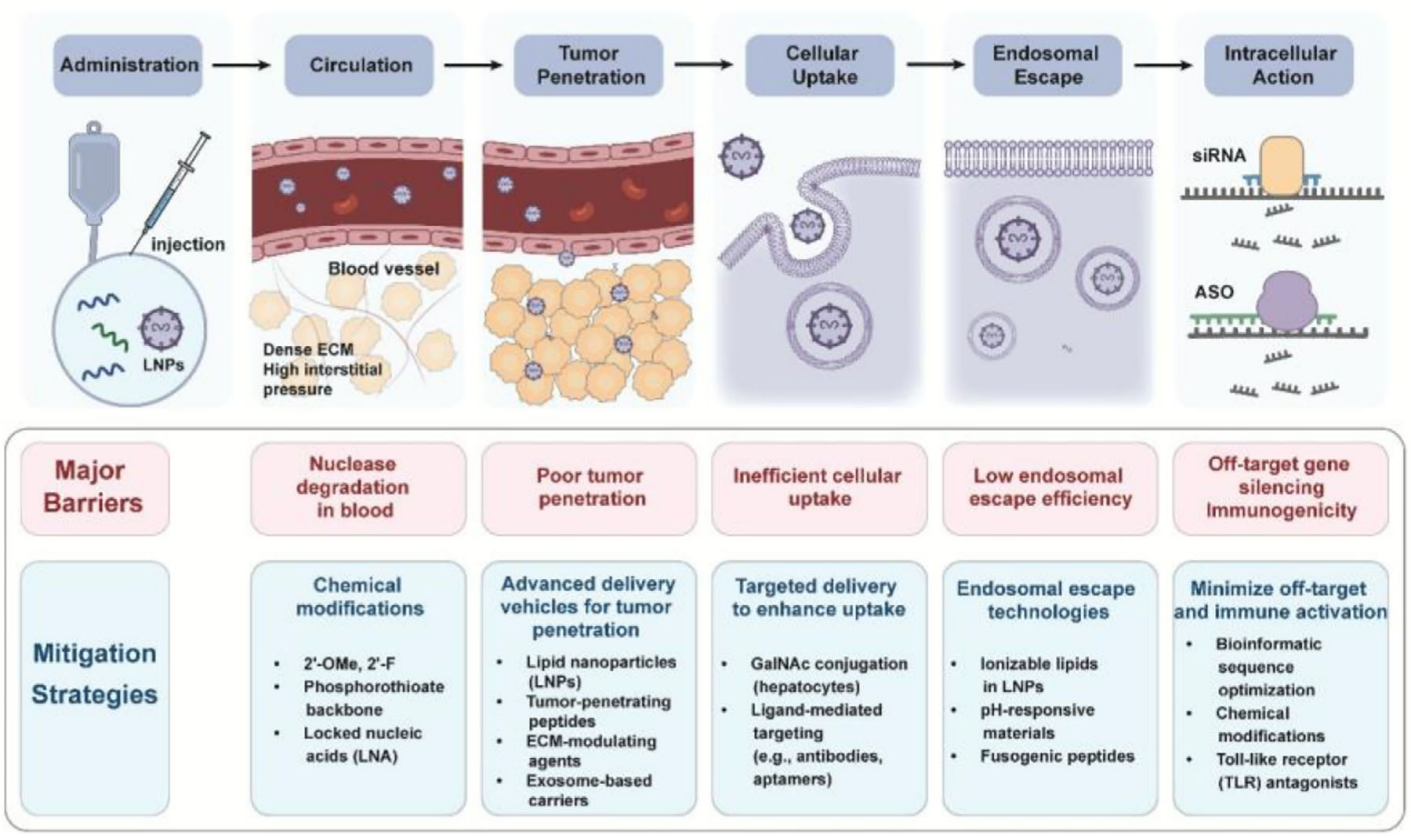
Safety and delivery barrier landscape of nucleic acid therapeutics for undruggable targets. Schematic overview of the major barriers and corresponding mitigation strategies in nucleic acid therapeutics. Barriers are shown in red boxes, including systemic degradation by nucleases, poor tumor penetration due to dense extracellular matrix and high interstitial pressure, inefficient cellular uptake, low endosomal escape efficiency, off‐target gene silencing, and immunogenicity. Mitigation strategies are shown in blue boxes, including chemical modifications, lipid nanoparticles, exosome‐based carriers, GalNAc conjugation, and bioinformatic sequence optimization.

Safety issues associated with nucleic acid therapeutics primarily involve immunogenicity, off‐target effects, and potential toxicity. Immunogenicity is often triggered by unmethylated CpG motifs within the sequence, which can be effectively mitigated through comprehensive chemical modifications (e.g., 2’‐O‐methyl, 2’‐fluoro, morpholino) [259]. Off‐target effects arise from partial sequence complementarity with non‑target mRNAs. Improved bioinformatic algorithms for rigorous off‑target prediction, combined with whole‑transcriptome screening, can significantly optimize sequence design and enhance specificity [260]. Additionally, specific delivery systems may introduce tissue‑specific toxicities. For example, the efficient hepatic accumulation of GalNAc‑based delivery systems may lead to unexpected hepatotoxicity following target gene knockdown, necessitating integrated pharmacological and toxicological assessments during drug design [261]. Additionally, for physiologically essential targets such as MYC, systemic inhibition carries a substantial risk of on target toxicity in normal proliferating tissues. Currently, no nucleic acid drug targeting Ras, MYC, or p53 has received FDA approval, and most evidence remains from preclinical models or early phase trials. The major adverse effects and corresponding mitigation strategies are summarized in Table 3, and an overview of safety and delivery barriers is presented in Figure 6.

**TABLE 3:** Potential adverse effects of nucleic acid therapeutics and corresponding mitigation strategies.

ADVERSE EFFECT CATEGORY	MECHANISM	CLINICAL MANIFESTATION	MITIGATION STRATEGY
IMMUNOGENICITY (INNATE)	Unmethylated CpG motifs activate TLR7/8/9	Cytokine release syndrome, flu like symptoms	Chemical modification (2'‐O‐Me, 2'‐F), sequence optimization, CpG removal
IMMUNOGENICITY (ADAPTIVE)	Peptide presentation from translated mRNA	Anti drug antibodies, reduced efficacy	Pseudouridine incorporation, purification of double stranded RNA contaminants
OFF‐TARGET GENE SILENCING	miRNA like partial complementarity with unintended mRNAs	Unpredicted phenotypes, potential toxicity	Rigorous bioinformatic screening, whole transcriptome off‐target analysis, locked nucleic acid incorporation
ON TARGET TOXICITY IN NORMAL TISSUES	Silencing or restoration of genes with physiological functions	MYC: gastrointestinal toxicity, myelosuppression; p53: tissue apoptosis	Tissue specific delivery (GalNAc, tumor targeted LNPs), local administration, dose optimization
DELIVERY VEHICLE TOXICITY	Cationic lipid induced complement activation	Infusion reactions, hepatotoxicity, pro‐inflammatory cytokine release	Ionizable lipid design, PEGylation, steroid co formulation, biodegradable lipids
NUCLEASE DEGRADATION	Bloodstream nuclease activity	Reduced efficacy, need for higher dosing	Chemical backbone modifications (phosphorothioate), protective nanoparticle encapsulation

### 5.3 Manufacturing and Cost

Scaling up the synthesis and purification of nucleic acid therapeutics from the laboratory to clinical application remains technically challenging [262]. The solid‑phase synthesis and downstream purification of long oligonucleotide sequences are complex processes that demand high technical standards to ensure batch‑to‑batch consistency [263]. Furthermore, the high development and production costs of personalized nucleic acid therapies designed based on individual mutation profiles represent a significant barrier to widespread adoption [264]. Driving innovation and standardization in manufacturing technology is crucial for reducing overall costs and improving the accessibility of these therapies.

## 6 Emerging Technologies and Future Perspectives

### 6.1 CircRNA Technology

With advances in basic research and technological innovation, the field of nucleic acid therapeutics is witnessing multiple promising new directions. Among these, circRNA technology stands out due to its covalently closed circular structure, which confers a significantly longer in vivo half‐life compared to linear RNAs by resisting exonuclease‐mediated degradation [265]. These molecules can serve as expression platforms for sustained therapeutic protein production or act as molecular sponges to sequester oncogenic miRNAs [266, 267, 268, 269]. The prolonged duration of action makes circRNA particularly well suited for long‐term modulation of oncogenic signaling networks where chronic target suppression is required [270, 271]. Recent engineering advances have enabled efficient circRNA synthesis and purification, paving the way for their evaluation in preclinical cancer models.

### 6.2 MRNA‐Based Protein Replacement Therapy

mRNA technology has expanded from preventive vaccines to enable in vivo expression of therapeutic proteins [272]. For loss‐of‐function undruggable targets such as mutant p53 that cannot be directly activated by small molecules, mRNA encoding wild‐type p53 can be delivered via lipid nanoparticles to restore tumor suppressor function. Similarly, mRNA encoding pro‐apoptotic factors or immunomodulatory cytokines offers strategies to bypass defective signaling pathways [273]. The flexibility of mRNA design allows rapid iteration and personalization based on patient‐specific mutation profiles, though challenges remain in achieving sufficient and sustained protein expression levels while minimizing immunogenicity.

### 6.3 CRISPR‐Based Gene Editing

Gene editing tools such as CRISPR Cas9 offer the potential for permanent genomic correction of disease‐driving mutations [274]. Unlike transient silencing approaches, CRISPR‐mediated editing can permanently disrupt mutant alleles or correct loss‐of‐function mutations. For KRAS‐driven cancers, CRISPR strategies have been developed to specifically target mutant alleles while sparing wild‐type Ras, addressing a key limitation of conventional approaches. However, clinical translation faces substantial challenges, including safe and efficient delivery of Cas9 and guide RNA components, off‐target editing at unintended genomic sites, and ethical considerations surrounding permanent genetic modification [275, 276]. Advances in lipid nanoparticle and viral vector delivery systems are beginning to address these barriers.

### 6.4 Artificial Intelligence Driven Design

Artificial intelligence is transforming the discovery and development pipeline for nucleic acid therapeutics [277]. By utilizing machine learning to predict RNA secondary and tertiary structures, optimize oligonucleotide sequences for specificity and potency, and design tissue targeting ligands de novo, AI accelerates candidate optimization and improves success rates [278]. Deep learning models trained on large‐scale oligonucleotide activity datasets can now predict off‐target hybridization risks and immunogenic potential before synthesis, enabling rational design of safer therapeutics [279, 280]. These computational tools provide a powerful platform for systematically tackling complex undruggable targets that have resisted traditional drug discovery approaches.

### 6.5 Combination Therapy Strategies

The strategic combination of nucleic acid therapeutics with existing therapies represents a key future direction. siRNA‐mediated knockdown of MYC can remodel the tumor microenvironment to enhance the efficacy of immune checkpoint inhibitors by reducing immunosuppressive factors and promoting T cell infiltration [193]. Similarly, inhibiting specific resistance genes using ASOs or siRNAs can reverse tumor resistance to conventional chemotherapy or targeted agents [281]. For KRAS mutant cancers, combining KRAS targeting nucleic acid drugs with CDK4/6 inhibitors or MEK inhibitors has shown synergistic effects in preclinical models [281]. These combination strategies hold promise for overcoming the limitations of monotherapies through coordinated multi‐pathway intervention, thereby improving overall therapeutic outcomes.

### 6.6 Future Perspectives on Clinical Translation

Looking forward, several key directions will shape the clinical translation of nucleic acid therapeutics for undruggable targets. First, continued improvement of delivery systems is essential, with particular emphasis on developing extrahepatic targeting capabilities beyond the liver. Second, safety optimization through chemical modification and sequence design will expand the therapeutic window. Third, the identification of predictive biomarkers for patient selection will enable precision application of these therapies to individuals most likely to benefit. Fourth, the integration of nucleic acid drugs into multimodal treatment regimens will likely be required for durable responses, given the complexity of cancer biology. While no nucleic acid drug targeting Ras, MYC, or p53 has yet received regulatory approval, the accumulating preclinical and early clinical evidence suggests that these challenges are solvable. The field is moving toward a future where undruggable is redefined as addressable through nucleotide‐based intervention.

## 7 Conclusion and Outlook

For decades, targets such as Ras, MYC, and p53 have posed significant challenges in drug discovery due to the lack of binding pockets suitable for traditional small molecules. The emergence of nucleic acid therapeutics, which shifts the intervention level from the protein itself to its mRNA transcript, has introduced a revolutionary paradigm for tackling these “undruggable” targets. This review systematically outlines how nucleic acid therapeutics, such as ASOs and siRNAs, achieve precise intervention against these refractory targets through mechanisms including post‑transcriptional gene silencing, translational suppression, splicing modulation, and the targeting of upstream regulatory networks, demonstrating their distinct advantages over conventional approaches.

The selection of Ras, MYC, and p53 as the focal targets of this review is justified not only by their long‑standing status as paradigms of undruggability but also by the substantial preclinical and clinical evidence that has accumulated for nucleic acid‑based interventions against them. Of note, similar strategies have recently shown promising results against other historically undruggable oncoproteins. For β‑catenin, lipid nanoparticle encapsulated siRNA targeting CTNNB1 induced tumor regression and immune reprogramming in hepatocellular carcinoma models [33]. For STAT3, a CpG‑conjugated antisense oligonucleotide combined with PD‑1 blockade achieved long‑term survival in glioblastoma [282]. For PTEN, lung‑targeted lipid nanoparticle delivered PTEN mRNA restored tumor suppressor expression and sensitized tumors to anti‑PD‑1 therapy, and a transdermal formulation also inhibited melanoma growth [283, 284]. For NF‑κB, peptide nanoparticle delivered siRNA targeting the c‑Rel subunit reduced tumor growth and enhanced CD8^+^ T cell responses [285]. These emerging examples further support the broad applicability of the nucleic acid therapeutic paradigm. Nevertheless, Ras, MYC, and p53 remain the most extensively validated models for mechanistic elucidation and translational roadmap design, which is the central focus of this review.

It is important to recognize, however, that nucleic acid therapeutics have not yet fully solved the undruggable problem. They provide a conceptually feasible path toward redefining druggability, but major challenges remain. The efficiency and specificity of delivery systems continue to be a major bottleneck for clinical translation, and concerns regarding immunogenicity, off‑target toxicity, and manufacturing processes require ongoing attention. In the future, with rapid advances in novel delivery carriers (e.g., engineered LNPs, exosomes), circRNA technology, mRNA encoding platforms, and AI‑driven rational design, nucleic acid therapeutics are expected to achieve significant breakthroughs in tissue targeting, stability, and safety. Furthermore, combining nucleic acid therapeutics with existing targeted treatments, immunotherapies, and other strategies to develop multi‑pathway synergistic regimens represents a highly promising direction.

In summary, nucleic acid therapeutics are reshaping the landscape of drug development for traditionally “undruggable” targets in unprecedented ways. As key technologies continue to advance, these therapies hold great promise for bringing new hope to patients with cancers, genetic disorders, and other major diseases, while continually expanding the frontiers of precision medicine.

## Author Contributions

F.X., K.W. and K.L. made contributions to conception and study supervision and searched data for this article. All authors contributed substantially to the discussion of the content, wrote the manuscript, and reviewed the manuscript before submission. All authors read and approved the final manuscript.

## Conflicts of Interest

The authors declare no conflict of interest.

## Data Availability

The data that support the findings of this study are available in the supplementary material of this article.
